# Low Pathogenic Avian Influenza A (H7N2) Virus Infection in Immunocompromised Adult, New York, USA, 2003

**DOI:** 10.3201/eid1807.111913

**Published:** 2012-07

**Authors:** Belinda Ostrowsky, Ada Huang, William Terry, Diane Anton, Barbara Brunagel, Lorraine Traynor, Syed Abid, Geraldine Johnson, Marilyn Kacica, Jacqueline Katz, Lindsay Edwards, Stephen Lindstrom, Alexander Klimov, Timothy M. Uyeki

**Affiliations:** Westchester County Department of Health, New Rochelle, New York, USA (B. Ostrowsky, A. Huang, W. Terry);; Westchester County Department of Laboratories and Research, Valhalla, New York, USA (D. Anton, B. Brunagel, L. Traynor, S. Abid);; New York State Department of Health, Albany, New York, USA (G. Johnson, M. Kacica);; and Centers for Disease Control and Prevention, Atlanta, Georgia, USA (J. Katz, L. Edwards, S. Lindstrom, A. Klimov, T.M. Uyeki)

**Keywords:** Avian influenza, H7N2, low pathogenicity, HIV, lower respiratory infection, adult, New York, immunocompromised, LPAI, United States, influenza, respiratory infections, influenza A

## Medscape CME Activity

Medscape, LLC is pleased to provide online continuing medical education (CME) for this journal article, allowing clinicians the opportunity to earn CME credit.

This activity has been planned and implemented in accordance with the Essential Areas and policies of the Accreditation Council for Continuing Medical Education through the joint sponsorship of Medscape, LLC and Emerging Infectious Diseases. Medscape, LLC is accredited by the ACCME to provide continuing medical education for physicians.

Medscape, LLC designates this Journal-based CME activity for a maximum of 1 *AMA PRA Category 1 Credit(s)*^TM^. Physicians should claim only the credit commensurate with the extent of their participation in the activity.

All other clinicians completing this activity will be issued a certificate of participation. To participate in this journal CME activity: (1) review the learning objectives and author disclosures; (2) study the education content; (3) take the post-test with a 70% minimum passing score and complete the evaluation at www.medscape.org/journal/eid; (4) view/print certificate.

**Release date: June 13, 2012; Expiration date: June 13, 2013**

## Learning Objectives

Upon completion of this activity, participants will be able to:

- Distinguish the usual severity of infections with LPAI
- Analyze the differential diagnosis for patients presenting with LPAI infection
- Evaluate the epidemiology of LPAI
- Assess other clinical characteristics of LPAI infection

## CME Editor

**P. Lynne Stockton, VMD, MS, ELS(D),** Technical Writer/Editor, *Emerging Infectious Diseases. Disclosure: P. Lynne Stockton, VMD, MS, ELS(D), has disclosed no relevant financial relationships.*

## CME Author

**Charles P. Vega, MD,** Health Sciences Clinical Professor; Residency Director, Department of Family Medicine, University of California, Irvine. *Disclosure: Charles P. Vega, MD, has disclosed no relevant financial relationships.*

## Authors

*Disclosures:* ***Belinda Ostrowsky, MD, MPH; William Terry, MD, MPH; Diane Anton, MS, M(ASCP); Barbara Brunagel, MS; Lorraine Traynor, BS; Syed Abid, PhD; Geraldine Johnson, MS; Marilyn Kacica, MD, MPH; Stephen Lindstrom, PhD; Alexander Klimov, PhD;*** *and* ***Timothy M. Uyeki, MD, MPH, MPP,*** *have disclosed no relevant financial relationships.* ***Ada Huang, MD,*** *has disclosed the following relevant financial relationships: owns stock, stock options, or bonds from Merck, Pfizer.* ***Jacqueline Katz, PhD,*** *has disclosed the following relevant financial relationships: received grants for clinical research from GlaxoSmithKline for research not related to the current study; received grants for preclinical research from Colby Pharmaceuticals (formerly Juvaris Bio Therapeutics) for research not related to the current study.* ***Lindsay Edwards*** *has disclosed the following relevant financial relationships: spouse owns GlaxoSmithKline stock.*
