# Supplementary material for: Low Pathogenic Avian Influenza A (H7N2) Virus Infection in Immunocompromised Adult, New York, USA, 2003
Source: Emerg Infect Dis. 2012 Jul;18(7):1128–31. doi: 10.3201/eid1807.111913 (PMC3376803; doi:10.3201/eid1807.111913)
Supplement: Related texts

# Brief report: Low Pathogenic Avian Influenza A (H7N2) Virus Infection in Immunocompromised Adult, New York, USA, 2003

**DOI:** 10.3201/eid1807.111913

**Authors:** Belinda Ostrowsky, Ada Huang, William Terry, Diane Anton, Barbara Brunagel, Lorraine Traynor, Syed Abid, Geraldine Johnson, Marilyn Kacica, Jacqueline Katz, Lindsay Edwards, Stephen Lindstrom, Alexander Klimov, Timothy M. Uyeki

**Affiliations:** Westchester County Department of Health, New Rochelle, New York, USA (B. Ostrowsky, A. Huang, W. Terry);; Westchester County Department of Laboratories and Research, Valhalla, New York, USA (D. Anton, B. Brunagel, L. Traynor, S. Abid);; New York State Department of Health, Albany, New York, USA (G. Johnson, M. Kacica);; and Centers for Disease Control and Prevention, Atlanta, Georgia, USA (J. Katz, L. Edwards, S. Lindstrom, A. Klimov, T.M. Uyeki)

## Abstract

In 2003, infection with low pathogenic avian influenza A (H7N2) virus was identified in an immunocompromised man with fever and community-acquired pneumonia in New York, USA. The patient recovered. Although the source of the virus was not identified, this case indicates the usefulness of virus culture for detecting novel influenza A viruses.

Limited numbers of human infections with low pathogenic avian influenza A, subtype H7, viruses have been reported and attributed to recent exposure to infected poultry (*1**–**6*). Such infections generally resulted in clinically mild illness. We report a case of low pathogenic avian influenza (LPAI) A (H7N2) virus infection in an immunocompromised adult.

## The Study

On November 3, 2003, a 48-year-old man from the Caribbean sought care at an emergency department in Westchester County, New York, USA, after an episode of near syncopy; a 2–4 week history of feverishness, cough, fatigue, and myalgia; and a 10-pound weight loss over 2 months. He had lived in the United States since 1987 and had no known medical conditions. A month earlier, he had been evaluated at a clinic, and an oral antimicrobial drug was prescribed for possible pneumonia. Eight days before the emergency department admission reported here, he had sought emergency care for unilateral conjunctivitis, eye pain, and blurred vision; the diagnosis was corneal abrasion.

Physical examination on November 3, 2003, found that the patient was afebrile, weak, and mildly tachypneic (respiratory rate 18–26 breaths/minute, room air oxygenation saturation 98%) with bibasilar inspiratory rales. Pertinent laboratory findings included mild anemia and thrombocytopenia (hemoglobin 11.9 g/dL, platelets 107 × 10^9^/L, leukocytes 8.0 × 10^9^ cells/L [52% lymphocytes]), mildly elevated hepatic transaminases (aspartate aminotransferase 116 U/L, alanine aminotransferase 87 U/L), and elevated creatine kinase (1,844 U/L). A chest radiograph showed a right hilar density and left lower lobe infiltrates; computed tomographic scan of the chest and abdomen showed bilateral micronodular opacities with right perihilar infiltrates and lymphadenopathy. The patient was admitted for community-acquired pneumonia and received intravenous gatifloxicin.

A tuberculin skin test was reactive (20-mm induration). HIV ELISA/Western blot test results were positive (HIV test result from 3 years earlier was reportedly negative), and CD4 count was 300 cells/μL. Treatment was switched to rifampin, isoniazid, pyrazinamide, ethambutol, and pyridoxine. Bronchoalveolar lavage (BAL) performed on November 7 yielded influenza A virus by tissue cell culture at the Westchester County Department of Laboratories and Research and was negative for *Pneumocystis* spp., *Legionella* spp., and other bacterial or viral pathogens. A second BAL and biopsy performed later during hospitalization to evaluate adenopathy indicated inflammation without definitive pathology. The lower respiratory tract disease improved after 13 days, and the patient was empirically prescribed tuberculosis treatment (directly observed therapy) and discharged while mycobacterial culture results were pending. After 8 weeks, mycobacterial culture of the BAL specimen was negative for *Mycobacterium tuberculosis* but yielded *M. avium* complex.

The patient lived in an apartment with his wife and 4 children, none of whom were sick during his illness. He denied recent travel and had not traveled outside the United States for 4 years. He worked in a cafeteria as a dishwasher and handled food and garbage until 1 month before hospitalization. He denied any known risk factors for HIV infection.

The influenza A virus isolate was difficult to grow in culture, reacted minimally with antiserum to hemagglutinin H1, and was sent to the Centers for Disease Control and Prevention (CDC) for further characterization. On March 19, 2004, CDC reported that the influenza isolate, designated A/New York/107/2003, was an LPAI A (H7N2), not subtype H1N1, virus.

An epidemiologic investigation was initiated by the Westchester County Department of Health. During 3 interviews (with a Creole interpreter), the patient denied any exposure to live or dead poultry, wild birds, or bird feces. No live poultry markets or poultry were found on the surrounding property or in the neighborhood.

Serum samples obtained during the patient’s hospitalization on November 5, 2003, and on April 4, 2004, were tested at CDC by microneutralization assay with the LPAI A (H7N2) virus from the patient. The acute-phase serum sample was negative (titer 10), but the convalescent-phase serum sample was positive (influenza [H7N2] virus neutralizing antibody titer 80), indicating seroconversion and evidence of infection with LPAI A (H7N2) virus. A confirmatory Western blot assay detected H7 hemagglutinin-specific antibody in the convalescent-phase serum sample. Testing of paired serum samples by ELISA demonstrated a 16-fold rise in H7 hemagglutinin-specific IgG. Serum samples collected from the patient’s wife and 3 of the children on April 4, 2004, were seronegative for influenza A (H7N2) neutralizing antibodies.

## Conclusions

In this immunocompromised man with pneumonia, the contribution of influenza A (H7N2) virus infection to his lower respiratory tract disease is unclear. The diagnosis of influenza A (H7N2) virus infection was not made until long after the patient had been discharged, and no influenza antiviral treatment was administered. The patient’s history and clinical findings were consistent with HIV and community-acquired pneumonia with possible clinical response to the antimicrobial drug therapy or improvement of self-limiting viral pneumonia. In patients with HIV infection, *M. avium* complex is often detected as an indolent pathogen, especially associated with disseminated disease in patients with advanced AIDS; clinical resolution usually requires prolonged multidrug treatment (*7*). Isolation of influenza A (H7N2) virus from a BAL specimen and resolution of lower respiratory tract disease during hospitalization suggest that this infection might have contributed to the pulmonary disease. The clinical spectrum of human infection with LPAI viruses, including subtype H7, is generally mild, ranging from conjunctivitis to influenza-like illness (*1**–**6*), although hospitalization of patients with influenza A (H7N2) virus infection has been reported (*6*).

Conjunctival infection with influenza A subtype H7 viruses in persons with conjunctivitis has been confirmed by reverse transcription PCR or virus isolation (*1**–**3**,**5*). The patient initially reported ophthalmologic symptoms. Because conjunctival specimens were not available for virus testing, the role of influenza A (H7N2) virus in the conjunctivitis is unknown. However, intraocular inoculation of mice with the influenza A (H7N2) virus from the patient (A/New York/107/2003) did not result in infection, and the virus replicated at relatively low levels in murine corneal epithelial cells ex vivo (*8**,**9*). Intranasal inoculation with the LPAI A (H7N2) virus caused respiratory symptoms, elevated mean lung titers, and cytokine increases in mice; among ferrets, the virus replicated efficiently in the upper respiratory tract and was transmissible through direct contact (*8**,**10*).

The source of the patient’s infection with influenza A (H7N2) virus was not determined, although exposure to poultry was suspected. A limitation is that the investigations were conducted 5 months after the patient was hospitalized, after the influenza A isolate was identified as an LPAI A (H7N2) virus. LPAI A (H7N2) viruses have been documented among birds in live poultry markets in the northeastern United States, including New York (*11**–**13*). Such viruses have receptor-binding properties consistent with receptors found in the human upper respiratory tract (*10*).

This case of LPAI A (H7N2) virus infection was detected through influenza virus surveillance of specimens submitted from outpatients and hospitalized patients to the Westchester County Department of Laboratories and Research and illustrates the value of virus culture for detection of human infections with novel influenza A viruses, which are nationally notifiable. For the patient reported here, neither seasonal influenza nor zoonotic influenza was suspected. Whether HIV infection might have made the patient more susceptible to lower respiratory tract infection with LPAI A (H7N2) virus is unknown, but a case of pulmonary infection with LPAI A (H9N2) virus in an immunosuppressed adult has been reported (*14*). A serologically confirmed case of LPAI A (H7N2) virus infection in the United States was associated with upper respiratory tract illness (*4*). Although information about the frequency of human infection with LPAI A H7 viruses is limited, 1 study reported antibody detection in 3.8% of exposed poultry workers after an outbreak of LPAI A (H7N3) virus infection among poultry in Italy (*15*). More information is needed to clarify the risk for LPAI A H7 virus infections among immunocompetent and immunocompromised persons.

## References

[1] Kurtz J, Manvell RJ, Banks J. Avian influenza virus isolated from a woman with conjunctivitis. Lancet. 1996;348:901–2. 10.1016/S0140-6736(05)64783-68826845

[2] Tweed SA, Skowronski DM, David ST, Larder A, Petric M, Lees W, Human illness from avian influenza H7N3, British Columbia. Emerg Infect Dis. 2004;10:2196–9. 10.3201/eid1012.04096115663860PMC3323407

[3] Skowronski DM, Tweed SA, Petric M, Booth T, Li Y, Tam T. Human illness and isolation of low-pathogenicity avian influenza virus of the H7N3 subtype in British Columbia, Canada. J Infect Dis. 2006;193:899–900. 10.1086/50021916479527

[4] Edwards LE, Terebuh P, Adija A, Rowe T, Kleene J, Hu-Primmer J. Serological diagnosis of human infection with avian influenza A (H7N2) virus. Presented at the International Conference on Emerging Infectious Diseases 2004, Atlanta, Georgia, February 22–March 3, 2004. Abstract 60, Session 44.

[5] Nguyen-Van-Tam JS, Nair P, Acheson P, Baker A, Barker M, Bracebridge S, ; Incident Response Team. Outbreak of low pathogenicity H7N3 avian influenza in UK, including associated case of human conjunctivitis. Euro Surveill. 2006;11:E060504.2 1681645610.2807/esw.11.18.02952-en

[6] Avian influenza A/(H7N2) outbreak in the United Kingdom. Euro Surveill. 2007;12:E070531.2.17868584

[7] Karakousis PC, Moore RD, Chaisson RE. *Mycobacterium avium* complex in patients with HIV infection in the era of highly active antiretroviral therapy. Lancet Infect Dis. 2004;4:557–65. 10.1016/S1473-3099(04)01130-215336223

[8] Belser JA, Lu X, Maines TR, Smith C, Li Y, Donis RO, Pathogenesis of avian influenza (H7) virus infection in mice and ferrets: enhanced virulence of Eurasian H7N7 viruses isolated from humans. J Virol. 2007;81:11139–47. 10.1128/JVI.01235-0717686867PMC2045519

[9] Belser JA, Wadford DA, Xu J, Katz JM, Tumpey TM. Ocular infection of mice with influenza A (H7) viruses: a site of primary replication and spread to the respiratory tract. J Virol. 2009;83:7075–84. 10.1128/JVI.00535-0919458003PMC2704783

[10] Belser JA, Blixt O, Chen LM, Pappas C, Maines TR, Van Hoeven N, Contemporary North American influenza H7 viruses possess human receptor specificity: implications for virus transmissibility. Proc Natl Acad Sci U S A. 2008;105:7558–63. 10.1073/pnas.080125910518508975PMC2396559

[11] Suarez DL, Spackman E, Senne DA. Update on molecular epidemiology of H1, H5, and H7 influenza virus infections in poultry in North America. Avian Dis. 2003;47(Suppl):888–97. 10.1637/0005-2086-47.s3.88814575082

[12] Senne DA, Suarez DL, Pedersen JC, Panigrahy B. Molecular and biological characteristics of H5 and H7 avian influenza viruses in live-bird markets of the northeastern United States, 1994–2001. Avian Dis. 2003;47(Suppl):898–904. 10.1637/0005-2086-47.s3.89814575083

[13] Bulaga LL, Garber L, Senne D, Myers TJ, Good R, Wainwright S, Descriptive and surveillance studies of suppliers to New York and New Jersey retail live-bird markets. Avian Dis. 2003;47(Suppl):1169–76. 10.1637/0005-2086-47.s3.116914575136

[14] Cheng VC, Chan JF, Wen X, Wu WL, Que TL, Chen H, Infection of immunocompromised patients by avian H9N2 influenza A virus. J Infect. 2011. Epub ahead of print. 10.1016/j.jinf.2011.02.00721356238

[15] Puzelli S, Di Trani L, Fabiani C, Campitelli L, De Marco MA, Capua I, Serological analysis of serum samples from humans exposed to avian H7 influenza viruses in Italy between 1999 and 2003. J Infect Dis. 2005;192:1318–22. 10.1086/44439016170747

# Brief report: Low Pathogenic Avian Influenza A (H7N2) Virus Infection in Immunocompromised Adult, New York, USA, 2003

**DOI:** 10.3201/eid1807.111913

**Authors:** Belinda Ostrowsky, Ada Huang, William Terry, Diane Anton, Barbara Brunagel, Lorraine Traynor, Syed Abid, Geraldine Johnson, Marilyn Kacica, Jacqueline Katz, Lindsay Edwards, Stephen Lindstrom, Alexander Klimov, Timothy M. Uyeki

**Affiliations:** Westchester County Department of Health, New Rochelle, New York, USA (B. Ostrowsky, A. Huang, W. Terry);; Westchester County Department of Laboratories and Research, Valhalla, New York, USA (D. Anton, B. Brunagel, L. Traynor, S. Abid);; New York State Department of Health, Albany, New York, USA (G. Johnson, M. Kacica);; and Centers for Disease Control and Prevention, Atlanta, Georgia, USA (J. Katz, L. Edwards, S. Lindstrom, A. Klimov, T.M. Uyeki)

## Medscape CME Quiz

## Earning CME Credit

To obtain credit, you should first read the journal article. After reading the article, you should be able to answer the following, related, multiple-choice questions. To complete the questions (with a minimum 70% passing score) and earn continuing medical education (CME) credit, please go to www.medscape.org/journal/eid. Credit cannot be obtained for tests completed on paper, although you may use the worksheet below to keep a record of your answers. You must be a registered user on Medscape.org. If you are not registered on Medscape.org, please click on the New Users: Free Registration link on the left hand side of the website to register. Only one answer is correct for each question. Once you successfully answer all post-test questions you will be able to view and/or print your certificate. For questions regarding the content of this activity, contact the accredited provider, CME@medscape.net. For technical assistance, contact CME@webmd.net. American Medical Association’s Physician’s Recognition Award (AMA PRA) credits are accepted in the US as evidence of participation in CME activities. For further information on this award, please refer to http://www.ama-assn.org/ama/pub/category/2922.html. The AMA has determined that physicians not licensed in the US who participate in this CME activity are eligible for *AMA PRA Category 1 Credits*™. Through agreements that the AMA has made with agencies in some countries, AMA PRA credit may be acceptable as evidence of participation in CME activities. If you are not licensed in the US, please complete the questions online, print the certificate and present it to your national medical association for review.

## Article Title: Low Pathogenic Avian Influenza A (H7N2) Virus Infection in Immunocompromised Adult, New York, USA, 2003

## CME Questions

**1. You are seeing a 48-year-old man who complains of 2 weeks of severe malaise, tactile fever, cough, and weight loss. You consider whether this patient has influenza. Which of the following statements best characterizes the majority of low pathogenic avian influenza (LPAI) viral infections?**

A. The mortality rate approaches 50%

B. Almost all cases are diagnosed in the hospital

C. Most infections are mild in nature

D. They are characterized by higher rates of secondary pneumonia compared with other influenza infections

**2. You want to offer targeted antimicrobial therapy for this patient. What was the patient in the current case study treated for originally?**

A. Community-acquired pneumonia

B. Primary influenza infection

C. Primary HIV infection

D. Tuberculosis

**3. The patient is confirmed to have infection with LPAI. How did the patient in the current case study acquire LPAI?**

A. Keeping pigeons above his home

B. Working in a poultry processing plant

C. Direct contact with infected coworkers

D. Unknown

**4. What else should you consider regarding the current case study as you treat this patient with LPAI infection?**

A. The presence of conjunctivitis suggests something different from infection with H7 subtype viruses

B. Concomitant HIV infection has been definitively associated with an increased risk of lower respiratory tract infection with H7N2 virus

C. The case patient recovered without specific treatment for influenza

D. The influenza A isolate grew rapidly in culture

## Activity Evaluation

**Table:** 

1. The activity supported the learning objectives.				
Strongly Disagree				Strongly Agree
1	2	3	4	5
2. The material was organized clearly for learning to occur.				
Strongly Disagree				Strongly Agree
1	2	3	4	5
3. The content learned from this activity will impact my practice.				
Strongly Disagree				Strongly Agree
1	2	3	4	5
4. The activity was presented objectively and free of commercial bias.				
Strongly Disagree				Strongly Agree
1	2	3	4	5
